# From Childhood Experiences to Social Media Addiction: Unraveling the Impact on Adolescents

**DOI:** 10.3390/children12030385

**Published:** 2025-03-19

**Authors:** Demet Aydın, Halide Bengü Göncü

**Affiliations:** 1 Department of Child Development, University of Health Sciences, Istanbul 34668, Türkiye; 2 Preschool Education, Ministry of National Education of Türkiye, Istanbul 34668, Türkiye; halideuzel@marun.edu.tr

**Keywords:** adolescents, childhood experiences, social media addiction, screen time

## Abstract

Social media usage among adolescents has significantly increased in recent years, playing a crucial role in contemporary youth culture. The increasing adoption of the internet across all age groups has led to numerous benefits, but also concerns regarding its misuse, particularly among adolescents. However, alongside these benefits, problems related to internet misuse have also escalated. **Background/Objectives**: A review of the literature reveals a lack of studies examining the long-term effects of childhood experiences on later social media addiction. This study aims to investigate the impact of childhood experiences on social media addiction during adolescence. **Methods**: This study employs a relational survey model, a quantitative research method. Data were collected using the Childhood Experiences Scale and the Social Media Addiction Scale for Adolescents. This study’s participants included 371 adolescents attending secondary education institutions under the Ministry of National Education of Türkiye during the 2024–2025 academic year. Participants were selected through simple random sampling. Relationship and impact tests were used to analyze the data. **Results**: This study found that adolescents with adverse school experiences exhibited social media addiction. A significant relationship was found between adverse school experiences and the time spent on social media. Furthermore, in terms of the gender variable, it was found that females scored significantly higher on the Social Media Addiction Scale for Adolescents compared to males. **Conclusions**: Based on the results of this study, it is observed that adverse experiences encountered in children’s school life may influence social media addiction in their future lives. In future studies, researchers may identify different childhood experiences that impact social media addiction.

## 1. Introduction

Nowadays, social media usage among adolescents has significantly increased. Especially after the pandemic, the frequency of social media use among adolescents has increased [1]. For this reason, social media plays a significant role in contemporary youth culture. According to data from the American Academy of Child and Adolescent Psychiatry (AACAP), 90% of adolescents aged 13–17 use social media. Additionally, 75% of adolescents have at least one active social media profile, and 51% visit at least one social media site daily [2]. According to the Household Information Technology Usage Survey conducted by the Turkish Statistical Institute (TÜİK), the internet usage rate among children aged 6–15 was 82.7% in 2021, increasing to 91.3% in 2024. Similarly, for individuals aged 16–74, internet usage was 85.0% in 2022, 87.1% in 2023, and 88.8% in 2024 [3]. All these statistics support the increasing trend of social media usage among adolescents.

There are various social media platforms designed and adapted for different purposes and target groups, such as Twitter, Facebook, and LinkedIn [4]. Among adolescents, highly visual social media platforms (e.g., Instagram, Snapchat) are particularly popular. These social media platforms primarily focus on sharing user-generated visual content (e.g., images, short videos) and allow users to modify visual content using filters [5]. It is important to explore what motivates adolescents to engage with highly visual social media platforms [6]. However, due to platform updates and changes in user demographics, the distinction between target audiences and user groups may not always be sharply defined. This study may be significant in understanding the increase in adolescents’ social media usage, as observed in the statistics above, and identifying the factors contributing to this rise. Most studies in the international literature have drawn attention to the potential negative consequences of social media use. While these studies focus on such negative outcomes [7,8], the relationship between early childhood experiences and these outcomes has not been adequately explored.

Early childhood is a critical period in which children experience constant change and development across all developmental domains [9]. Critical periods are defined as specific phases in an individual’s developmental process during which a particular event or stimulus has the most significant impact [10]. This phase is crucial because it is when children acquire the fundamental knowledge and skills they will use throughout their lives [11]. The potential for an individual’s genetic inheritance to either regress or develop depends on the impact of childhood experiences [12]. Environmental, emotional, and social factors during childhood shape individuals’ behavioral patterns [13]. Although families have a significant influence, in some cases, they fail to provide the ideal environment for the child’s protection, personality development, and necessary attention and support. The lack of protection, personality development, and essential care in early childhood can impact the child’s future experiences [14]. Since early childhood experiences profoundly affect later life, they may leave deep imprints on social media usage during adolescence. Identifying the relationship between childhood experiences and social media addiction on adolescents can help highlight the importance of early childhood in addressing the increasing and potentially harmful use of social media. This understanding may enable the development of preventive interventions. Influenced by adverse life experiences, adolescents may turn to excessive social media use to escape the emotions associated with these experiences [15].

Recent studies on adolescents’ social media use focus on its immediate effects on individuals. When reviewing previous studies, some research highlights social media’s negative impact on adolescents’ psychology [16,17,18,19], body image disorders [5,20] and behavior [21,22,23]. Unlike these studies, one study conducted with university students revealed a relationship between adverse childhood experiences and mobile phone addiction [24]. This study specifically examined adverse childhood experiences, attachment anxiety, attachment avoidance, and interpersonal relationship variables. Studies on childhood experiences have found that adverse experiences during childhood negatively affect adolescents both personally and psychologically [19,25,26,27]. In one study, childhood traumas and parental attitudes were found to influence depression and attachment levels on adolescents [25]. Another study examined the relationship between childhood traumas and mental health on adolescents displaying risky behaviors. The findings indicated that emotional neglect in childhood was associated with depression in adolescence [19]. In addition to these studies, Badenes-Ribera et al. [28] demonstrated in their research that attachment to parents during childhood (particularly parental alienation) is associated with social media addiction, specifically Facebook addiction. The study reveals that while peer attachment tends to be stronger in late adolescence, parental attachment in early adolescence plays a significant role in the increase in social media addiction. Building on these findings, it suggests that risky behaviors, such as social media addiction, may be linked to childhood experiences. Dangerous behaviors directly or indirectly affect young adolescents’ health, well-being, and lifestyles, potentially leading to negative consequences [29]. When reviewing studies that examine the relationship between childhood experiences and social media addiction in adolescents, research focuses on attachment styles [30,31] and childhood traumas [32]. These studies have identified a link between attachment styles, childhood traumas, and social media addiction.

Unlike previous research, the present study aims to contribute to the literature by examining the effects of adolescents’ subdimensions of family, school, and personal–social life on social media addiction. Prior research investigated the relationship between childhood experiences, happiness, and social media addiction among adolescents [33]. However, this study differed from others by including a sample group from different regions of Türkiye, considering various variables, and being based on a different theoretical framework. Therefore, this study expects to contribute to the literature by further elucidating the relationship between childhood experiences and social media addiction.

### Theoretical Framework

With the widespread use of the internet among preschool children, adolescents, and adults, it has been observed that it brings various positive contributions to human life. However, alongside these benefits, problems related to the misuse of the internet have also increased [7,8,34]. Individuals who experience unhappiness and depression tend to rely on the internet for social support, which, in turn, negatively affects their real-life interpersonal relationships and increases the risk of internet addiction [35]. Studies have supported the idea that risky behaviors on adolescents may be influenced by their childhood experiences. In this context, the theoretical framework of this study, which examines the relationship between adolescents’ childhood experiences and social media use, is based on the Social Network Theory [36,37,38,39] and the Compensatory Internet Use Theory [15].

Childhood experiences play a crucial role in shaping attitudes and behaviors during adolescence. Accessing information about individuals’ school life, family life, and/or personal–social life during childhood provides valuable insights into the root causes of problems [40]. Adverse childhood experiences contribute to emotional and behavioral issues during adolescence, with social media addiction emerging as one of these challenges [33]. In this study, social media addiction refers to an individual’s loss of control over social media activities and an inability to imagine life without social media [41]. It is hypothesized that the root cause of common adolescent problems, such as social media addiction, may be linked to childhood experiences.

The Social Network Theory fundamentally centers on the idea that individuals interact through relationships and cannot be viewed in isolation from their social environment. In this theory, networks consist of actors who establish relationships with one another, forming overall social structures and shaping interactions within these structures [36,39]. The structure of social interactions facilitates or restricts access to valuable resources, which may be related to work (e.g., task advice, feedback information) or personal support (e.g., emotional support, encouragement). According to the Social Network Theory, an individual’s position within a network is associated with performance-related outcomes [42]. The Social Network Theory posits that the most effective way to study a social group is to examine the network connections that involve interactions between members (actors) within the group [43]. An individual’s social network plays a crucial role in shaping their social media behavior. This influence can encourage addictive usage among individuals [4,44]. This study, therefore, aligns with the Social Network Theory and the Strength of Weak Ties Theory, which is embedded within it. The weakness of interpersonal networks can influence individuals to seek alternative social interaction methods, such as social media. These theories suggest that individuals with weaker social networks are more likely to rely on and gravitate toward weak ties in social media interactions [37,38]. In other words, childhood experiences are shaped by the interactions between these networks. When analyzed with the Compensatory Internet Use Theory [15], these theories provide a strong theoretical foundation for understanding the link between childhood experiences and social media usage. The Compensatory Internet Use Theory [15] highlights that individuals may use the internet as a coping mechanism to alleviate adverse life experiences and emotions. The central premise of this theory is that excessive internet use stems from an individual’s response to adverse life situations facilitated by digital applications. The Social Network Theory [36,37,38,39,42,43] may explain how childhood experiences are shaped through networks and highlights their impact on an individual’s development. Meanwhile, the Compensatory Internet Use Theory [15], suggests that these adverse experiences influence an individual’s digital behaviors. Together, these theories provide a foundational perspective on the potential relationship between childhood experiences and social media addiction.

Based on these theories, adolescents may turn to excessive social media use to respond to adverse childhood experiences. Therefore, this study aims to examine the impact of adolescents’ childhood experiences on social media addiction. A review of the literature reveals that research investigating the impact of childhood experiences and social media addiction among adolescents remains limited. Thus, by emphasizing the connection between childhood experiences and social media addiction, this study is expected to contribute to the existing body of research. Additionally, the findings of this study may serve as a guide for preventive interventions addressing the rapidly increasing prevalence in social media addiction.

This study aims to examine the impact of childhood experiences on social media addiction during adolescence. There are three main hypotheses in this study aimed at achieving this objective.

1. Adolescents’ adverse childhood experiences and social media addiction differ based on gender.
2. Adolescents’ adverse childhood experiences and social media addiction differ based on the duration of social media use.
3. Adolescents’ adverse childhood experiences, gender, and social media usage duration affect their social media addiction.

## 2. Materials and Methods

### 2.1. Subsection

The Cankırı Karatekin University Unit of Scientific Research and Ethical Review Board has approved the study protocol. The research was conducted using the relational survey model, one of the quantitative research methods. The relational survey model is a statistical approach used to determine the tendency or pattern of co-variation between two or more variables [45]. In this study, the relational survey model was chosen as it aims to reveal the impact of childhood experiences on social media addiction during adolescence.

### 2.2. Measurement Tools

In this study, the Childhood Experiences Questionnaire [40] and the Social Media Addiction Scale for Adolescents [41] were used as data collection instruments.

The Personal Information Form and the Adolescent-Family Consent Form were created to obtain information about adolescents based on variables determined by the researchers. This form includes questions regarding the student’s gender, school type, grade level, mother’s education level, and father’s education level. In accordance with ethical guidelines, parental consent was obtained before administering the form, and no questions related to the student’s identity were asked during this study.

The Childhood Experiences Questionnaire [40] consists of 12 items divided into three subdimensions: Family Life (4 items), School Life (4 items), and Personal–Social Life (4 items). The scale is assessed using a five-point Likert scale (“Always (1)”, “Often (2)”, “Sometimes (3)”, “Rarely (4)”, “Never (5)”). The score range for each subdimension is 4 to 20, with higher scores indicating a greater presence of adverse childhood experiences. Items 5 and 7 in the School Life subdimension are reverse-scored. During the scale development process, exploratory factor analysis (EFA), confirmatory factor analysis (CFA), and reliability analysis were conducted. As a result of EFA, the scale was identified as having three factors, consisting of Family Life (4 items), School Life (4 items), and Personal–Social Life (4 items). The factor loadings for each subdimension were found to range between 0.718 and 0.802 for Family Life, 0.508 and 0.843 for School Life, and 0.646 and 0.751 for Personal–Social Life. The CFA results confirmed that the model fit was at an excellent or acceptable level. When examining the reliability analysis results, the Cronbach’s alpha (Cr-α) coefficients were determined as 0.822 for Family Life, 0.770 for School Life, and 0.695 for Personal–Social Life. These findings indicate that the scale is valid and reliable in assessing childhood experiences.

The Social Media Addiction Scale for Adolescents [41] consists of a single factor with 9 items. The scale follows a five-point Likert structure (“Never (1)”, “Rarely (2)”, “Sometimes/Occasionally (3)”, “Frequently (4)”, “Always (5)”). The Social Media Addiction Scale for Adolescents presents participants with a wide range of platforms and includes examples covering various social media applications (MySpace, Facebook, Bebo, LinkedIn, Tumblr, Blogs, Instagram, WhatsApp, Viber, Line, Tango, Snapchat, Wikipedia, Apple iTunes Podcasts, Forums, YouTube, Twitter, etc.). The highest possible score on the scale is 45, while the lowest is 9. There are no reverse-scored items in the scale. To verify the accuracy of the single-factor structure determined through exploratory factor analysis (EFA), confirmatory factor analysis (CFA) was conducted. New data were collected to test the structure identified in the EFA, and the model fit indices [χ^2^/df = 2.694; GFI = 0.92; AGFI = 0.87; CFI = 0.95; RMR = 0.06; SRMR = 0.04] indicated that the structure demonstrated a good fit. As the scores obtained from the scale increase, it suggests a higher level of social media addiction among adolescents, whereas lower scores indicate a lower level of addiction. The Cronbach’s alpha (Cr-α) coefficient for the scale was calculated as 0.904, demonstrating high reliability.

### 2.3. Participants

The participants of this study consisted of 371 adolescents enrolled in secondary education institutions affiliated with the Ministry of National Education of Türkiye during the 2024–2025 academic year.

An analysis of the gender distribution of the adolescents participating in this study showed that 24.26% (n = 90) of the participants were male, while 75.74% (n = 281) were female. The demographic characteristics of the participants are presented in Table 1.

### 2.4. Data Collection and Analysis

During the data collection process, the necessary permissions for the research were first obtained. The simple random sampling method was used to select the participants. After obtaining the necessary research permits, cities from all seven regions of Türkiye were selected using the simple random sampling method. From these selected seven cities, seven different high schools were also chosen through simple random sampling. Meetings were held with school principals and district directorates of national education, and the required permissions were obtained. Subsequently, the scale form was prepared digitally and shared with high school teachers. The teachers were informed about this study, and they, in turn, informed the adolescents and their families, requesting them to complete the consent form. The scales were completed by adolescents in an average of 16 min. The data collection process was completed over a period of two months, from January to February 2025.

All data were recorded and analyzed using the SPSS 22.0.0.0 (Statistical Package for Social Sciences) for Windows 22 software. In the data analysis process, the assumptions that needed to be met were first tested to determine which statistical tests (parametric/non-parametric) would be applied. The normality of the distribution was assessed using the Kolmogorov–Smirnov test, as well as skewness and kurtosis values and histogram graphs, which are additional assumptions of normal distribution.

For comparisons between two independent groups, an Independent Samples *t*-test was used, while a one-way analysis of variance (ANOVA) was applied for comparisons among two or more unrelated groups. The relationships between numerical variables were examined using Pearson correlation analysis. To investigate the effect of the independent variable on the dependent variable, multiple regression analysis was conducted. A significance level of 0.05 was used as the criterion for interpreting the statistical significance of the obtained values.

Table 2 shows that the normality of the scale and subdimension scores were assessed using the Kolmogorov–Smirnov test and by examining skewness and kurtosis coefficients. For variables found to be significant in the Kolmogorov–Smirnov test (*p* < 0.05), if the skewness and kurtosis values were within the range of ±2.0 [46], this was interpreted as an indication that the values did not exhibit excessive deviation from normal distribution. Consequently, the analyses were conducted using parametric tests.

The reliability of the scale scores used in this study was tested using the Cronbach’s Alpha internal consistency test. The alpha coefficient is defined as the weighted average of the standard deviation, calculated by the ratio of the total variance of specific items in the scale to the overall variance [47]

The Cronbach’s Alpha coefficient is measured on a scale from 0 to 1, with reliability increasing as the value approaches 1 [48]. In this study, the family life subdimension had a Cronbach’s Alpha of 0.89, the school life subdimension had 0.75, the personal/social life subdimension had 0.86, and the social media addiction scale had 0.85. These findings indicate that the reliability coefficients were high.

## 3. Results

### 3.1. Gender

In the analysis conducted based on the gender variable, it was determined that the family life subdimension scores were 4.48 ± 0.72 for males and 4.32 ± 0.90 for females, with this difference found to be not statistically significant (t = 1.46; *p* > 0.05). Similarly, no significant differences were found between genders in the school life subdimension (t = −1.54; *p* > 0.05) and the personal/social life subdimension (t = 1.04; *p* > 0.05). However, in the Social Media Addiction Scale for Adolescents, the scores were 2.08 ± 0.81 for males and 2.35 ± 0.92 for females, and this difference was found to be statistically significant (t = −2.55; *p* < 0.05).

### 3.2. Time Spent on Social Media

The analysis revealed significant findings regarding social media usage and its impact. In the school life subdimension, individuals who used social media for 0–2 h per day had significantly higher scores compared to those who used it for 4–6 h per day (F = 4.29; *p* < 0.05).

Regarding the Social Media Addiction Scale for Adolescents, addiction scores increased as social media usage time increased. Individuals who used social media for 0–2 h per day had significantly lower addiction scores compared to all other groups (F = 38.09; *p* < 0.01). Additionally, those who used social media for 2–4 h per day had significantly lower addiction scores than those who used it for 4–6 h and more than 6 h per day. These analyses are presented in Table 3.

These results show that increased social media usage is associated with higher social media addiction scores, and excessive use negatively affects school life.

### 3.3. The Impact of Adverse Childhood Experiences and Certain Variables on Adolescents’ Social Media Addiction

A regression analysis was conducted to examine the effect of adverse childhood experiences on adolescents’ social media addiction. Table 4 shows that the regression analysis results indicated that the model explained 26.5% of the total variance in the Social Media Addiction Scale for Adolescents scores (Adjusted R^2^: 0.265). The model was found to be statistically significant (F(7.363) = 20.03; *p* < 0.01). When examining the effects of the independent variables on the Social Media Addiction Scale for Adolescents, it was determined that the family life subdimension (B = −0.06, *p* > 0.05) and the personal/social life subdimension (B = 0.04, *p* > 0.05) did not have a significant effect. However, the school life subdimension was found to be a significant positive predictor of social media addiction (B = −0.15, *p* < 0.01). This finding suggests that as the school life subdimension score increases, the level of social media addiction increases by approximately 0.15 times.

When evaluated in terms of the gender variable, it was found that females had significantly higher scores on the Social Media Addiction Scale for Adolescents compared to males (B = 0.27, *p* < 0.01). This finding indicates that females’ social media addiction scores were approximately 0.27 points higher than those of males.

Regarding the time spent on social media variable, when 0–2 h of usage was taken as the reference category, it was determined that individuals who used social media for 2–4 h per day had significantly 0.35 points higher addiction scores compared to the reference group (B = 0.35, *p* < 0.01). Similarly, those who used social media for 4–6 h per day had 0.84 points higher scores compared to the reference group (B = 0.84, *p* < 0.01). Furthermore, individuals who used social media for 6 h or more per day had 1.22 points higher addiction scores compared to the reference group (B = 1.22, *p* < 0.01).

These findings indicate that as social media usage duration increases, the level of social media addiction also increases significantly.

In conclusion, time spent on social media was identified as the strongest predictor variable in the model. Specifically, individuals who used social media for 6 h or more per day had 1.22 points higher scores on the Social Media Addiction Scale compared to those who used it for 0–2 h. Similarly, individuals who used social media for 4–6 h per day had 0.84 points higher scores, while those who used it for 2–4 h per day had 0.35 points higher scores compared to the reference group.

Gender was also found to be a significant predictor of social media addiction, with females scoring 0.27 points higher than males on the Social Media Addiction Scale. However, the family life and personal/social life subdimensions were found to have no significant effect on social media addiction.

## 4. Discussion

This study aimed to examine the impact of adverse childhood experiences on social media addiction among adolescents. In line with this objective, the analyses conducted indicate that adverse school childhood experiences influence social media addiction on adolescents.

### 4.1. Adolescents’ Adverse Childhood Experiences and Social Media Addiction and Gender

The girls participating in this study use social media more than boys. However, there is no difference between genders in terms of adverse childhood experiences. Deniz et al. [49] in their study examining high school students’ social media addictions, found that female students spend more time on social media than male students. Additionally, studies supporting these findings are available in the literature [50,51,52,53,54]. However, a study has revealed that women are more likely than men to develop social media addiction [55]. However, in a study examining social media addiction among university students, Baz [56] found that social media addiction does not differ based on gender. Çiftçi’s [57] study examining social media addiction among university students found that males are more addicted to social media than females. In another study investigating gender differences in social media use among university students and its effects on academic performance, it was revealed that male students are more addicted to social media, whereas female students’ academic performance is more negatively affected by social media use [58]. When these studies are examined, it can be stated that the relationship between social media addiction and gender is complex and may vary depending on different factors. Gender can influence the type and purpose of social media use, which in turn may affect addiction levels. In this regard, it can be said that this study has reached similar results to most studies in the literature. However, the effect of the gender variable on social media addiction is not definitive, as it may vary due to the characteristics of the participants in the studies (e.g., socio-economic status, peer relationships, social skill levels, parental attitudes, and parents’ educational backgrounds).

### 4.2. Adolescents’ Adverse Childhood Experiences and Social Media Addiction and the Duration of Social Media Use

According to the results of this study, there is a significant difference between negative school experiences and the duration of social media use. It has been revealed that as the negative school experience scores increase, the duration of social media use also increases. A review of the literature shows that there are various studies examining the relationship between internet use and academic performance. Duman’s [59] studies examining the impact of internet use on academic achievement found that internet use has a negative effect on academic performance. Another study revealed that spending excessive time on social media contributes to academic procrastination [60]. Daysal et al. [61] have determined that smartphone addiction leads to low academic achievement among adolescents. Another study found that social media use reduces productivity, negatively affects academic achievement, and leads to continuous media consumption [62]. A review of the studies suggests that excessive and unconscious use of social media may negatively affect individuals’ academic performance. In this regard, these findings can be considered parallel to the results of the present study.

When negative school experiences are mentioned, various adverse situations (e.g., peer bullying, lack of friendships, and adaptation problems) may come to mind. A review of recent studies highlights peer bullying as a prominent issue among these negative experiences. It has been determined that children who are exposed to peer bullying also have to cope with various other adverse situations [63,64,65,66]. A study revealed that some children aged 9–15 are exposed to peer bullying, while others engage in bullying, and both groups experience psychological adaptation problems. Additionally, it was found that these children are also at risk of internet addiction [67]. The results of this study can be interpreted as indicating a relationship between internet addiction and bullying. Supporting this finding, another study found a strong relationship between adolescents’ perceptions of the school climate, social media use, and peer bullying, with peer bullying playing a partial mediating role in this relationship [68]. Another study found that being exposed to peer bullying negatively affects adolescents’ self-perception and may lead to social media addiction [69]. Based on the conducted studies, it can be stated that peer bullying may lead to social media addiction [67,68,69,70]. This study also found that negative school experiences influence social media addiction. In this regard, the results can be considered supportive of each other. Given that social media addiction often leads to social isolation and negative psychological states, it is not surprising that these findings are consistent. Moreover, considering that the social media usage time of high school students participating in this study increases as their negative school experience scores rise, it can be stated that past negative school experiences also contribute to increased social media use. This finding highlights the unique aspect of this study compared to others. While previous studies have examined the relationship between current conditions, this study investigates the impact of past experiences on present behavior.

According to the results of this study, as the duration of social media use increases, social media addiction scores also increase. Tanrıverdi et al. [71] found in their study that as students’ levels of social media adoption increased, their year-end academic achievement and foreign language grades declined. Similarly, Bilgin [72] identified a strong relationship between social media addiction and daily social media usage duration. Deniz et al. [49] determined in their study that when the average daily time spent on the internet exceeded 30 min, high school students’ social media addiction increased gradually. Likewise, Uslu [53] found a positive and significant relationship between social media usage time and social media addiction. When comparing the research findings, it becomes evident that social media usage duration has a substantial impact on social media addiction. A similar conclusion was reached in this study, revealing that as social media usage duration increases, social media addiction scores also rise. A review of the literature indicates that this finding aligns with existing research, suggesting that spending excessive time on social media may lead to social media addiction.

### 4.3. Adolescents’ Adverse Childhood Experiences (School Life Subdimension) Affect Their Social Media Addiction

This study aims to examine the impact of high school students’ adverse childhood experiences on their social media addiction. In line with this objective, the analyses indicate that adverse childhood experiences, particularly negative school experiences, influence students’ social media addiction. A review of the literature reveals that Şanlı [73] identified positive childhood experiences as a significant predictor in reducing smartphone addiction. Similarly, Sheng et al. [32] in their study investigating the relationship between internet addiction and childhood trauma, found that such traumas increase the risk of internet addiction. Examining these results, it can be stated that negative experiences may increase individuals’ use of social media or the internet. In a sense, negative experiences can be considered a risk factor for social media addiction. This study also aligns with the existing literature, suggesting that negative school experiences pose a risk for social media addiction. A review of the literature reveals studies indicating that family life [74,75,76,77] and social life [78,79,80] influence social media addiction. The relevant literature suggests that individuals experiencing social isolation and negative family experiences are at a higher risk of social media addiction compared to others. However, such an effect is not observed in this study. This may be because this study’s findings are based on the responses of 371 high school students. Additionally, individuals’ susceptibility to negative experiences varies, which could also account for the difference in findings. This situation may have been influenced by various factors (e.g., socioeconomic status, parental attitudes, urban opportunities, peer relationships, and social skill levels). In this regard, future studies could examine the effects of these variables on social media addiction.

This study aimed to examine the impact of childhood experiences—a critical period for individual development—on the present situation. A review of the literature reveals that previous studies have primarily focused on the effects of individuals’ recent or current negative experiences on social media addiction. Additionally, a study investigating the relationship between social media addiction and depression found a significant positive correlation between the two variables [81]. Beyazgül-Tosun [82] examined the relationships between adolescents’ social media addiction, school engagement, and family life satisfaction. The findings indicate that as the level of school engagement decreases, social media addiction increases. This study also found that negative school experiences influence high school students’ social media addiction. In this regard, it can be stated that these two studies have reached similar conclusions. Similarly, a review of the literature reveals studies that, like this research, have identified a positive relationship between social media addiction and depression [83,84,85,86,87]. A review of the studies suggests that socially disadvantaged individuals are at a higher risk of social media addiction compared to others. Similarly, this study found that individuals with negative school experiences are more likely to develop social media addiction. In this regard, it can be stated that the findings of this study are consistent with previous research. Another study revealed that high school students with a higher risk of depression spend more time on social media than others [88]. A review of the literature reveals that longitudinal studies conducted with psychologically disadvantaged individuals have also found that these individuals exhibit higher levels of social media addiction [89,90,91]. Another study found that the unconscious use of social media can lead to eating disorders and psychiatric problems among adolescents [92]. Similarly, another study emphasized that the problematic use of social media platforms negatively affects children’s behaviors and eating habits [93]. Examining these findings, it can be stated that adverse conditions are positively associated with social media addiction. This study also revealed that high school students with negative school experiences have higher social media addiction scores. Furthermore, it was determined that negative school experiences influence high school students’ social media addiction. In this regard, the findings of this study align with the existing literature. Moreover, it stands out from other studies by specifically highlighting the impact of negative school experiences on social media addiction. This result underscores the significance of schools in individuals’ development and overall quality of life. However, this study found that negative experiences in the personal/social domain do not have an impact on social media addiction. In this regard, the findings differ from the studies. A review of the literature indicates that personal/social experiences are generally associated with social media addiction [94,95,96]. However, this study reached the opposite conclusion. In this regard, it can be stated that this research contributes to the existing literature. Additionally, the presence of different participants in each study, along with their unique experiences and characteristics, as well as the fact that analyses are conducted based on their responses at a given moment, may have led to this difference in findings.

## 5. Conclusions, Educational Remarks, and Limitations

**Figure 1:**
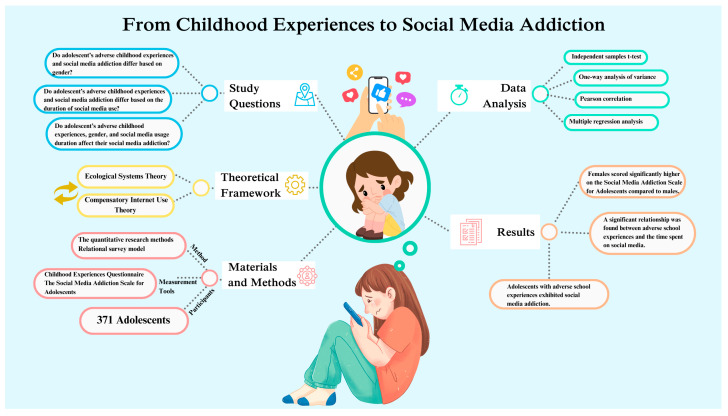
The research process, method, and important results.

This study concluded that negative school experiences influence adolescents’ social media addiction. Additionally, as shown in Figure 1, as the duration of social media use increases, social media addiction also rises. The results further indicate that female students in this study exhibit higher levels of social media addiction compared to male students. In light of these findings, it can be stated that school experiences have various effects on individuals’ lives, and negative experiences may lead to adverse outcomes. While the positive impacts of technology on our lives are undeniable, excessive and unregulated use without proper time management can negatively affect individuals. Furthermore, gender differences in social media addiction should be considered, as the type of social media platforms used may contribute to variations in addiction levels between males and females.

This study was conducted with 371 high school students, including 281 females and 90 males. The lower number of male participants presents a limitation for this research, which may have resulted from female students being more willing to participate. Future studies could address this limitation by ensuring a more balanced representation of male and female participants. In this study, the effects of gender, social media usage duration, and adverse childhood experiences on social media addiction were examined. Future research could further explore the influence of additional variables such as age, socioeconomic status, parental attitudes, parental education levels, family communication, participants’ leisure activities and hobbies, peer relationships, and social skills on social media addiction. Additionally, longitudinal studies could be conducted to examine the long-term effects of adverse childhood experiences on social media addiction.

Moreover, upcoming studies could employ more comprehensive and multidimensional scales to measure social media addiction. Unlike this study, which aimed to assess general social media usage habits without focusing on specific platforms, future research could investigate how different platforms serve distinct functions and cater to various demographic groups. For instance, highly visual platforms (e.g., Instagram, Snapchat) are more commonly used among adolescents compared to applications where visual content is less prominent. This trend may contribute to negative effects on body image perception and psychological well-being [5].

Negative school experiences can be seen as a collection of experiences that lead adolescents to feel lonely due to their weak social ties. According to Social Network Theory [36,39] and its sub-theory, the Strength of Weak Ties Theory [37,38], weak interpersonal connections may drive individuals toward alternative forms of social interaction, particularly social media. Similarly, the Compensatory Internet Use Theory [15] highlights that individuals may use the internet as a coping mechanism to alleviate negative life experiences and emotions. Given the findings of this study, it can be argued that these two theories find concrete application in practice. Adolescents’ insufficient social skills during their early school years, experiences of peer bullying, and difficulties in peer relationships may contribute to the development of social media addiction in later years.

This study’s results suggest that adverse school experiences during childhood may contribute to long-term patterns of social media dependency. The findings suggest that adolescents should be supported in their social media use. Promoting conscious social media usage, limiting screen time, providing adolescents with high-quality and safe activities where they can express themselves socially, and improving school experiences, particularly during primary and middle school years, may help reduce social media addiction among adolescents. Young individuals can be supported in developing hobbies that allow them to spend their free time in a meaningful and productive manner. Access to professional support services can be provided to enhance their social well-being. Additionally, parents can be offered guidance on conscious social media and technology use. Schools should regularly monitor the development of children’s social skills and expand intervention programs. When faced with negative situations such as bullying, effective measures should be taken to address the issue promptly and appropriately.

## Figures and Tables

**Table 1:** Distributions of Demographic Characteristics.

Variables	Groups	f	%
Mother’s educational level	High School Education or Lower	285	76.82
	High School	62	16.71
	University	24	6.47
Father’s educational level	High School Education or Lower	248	66.85
	High School	86	23.18
	University	37	9.97
Gender	Boy	90	24.26
	Girl	281	75.74
Time spent on social media	0–2 h	109	29.38
	2–4 h	107	28.84
	4–6 h	116	31.27
	6+ h	39	10.51

**Table 2:** Findings Related to the Normality Assumption and Summary Statistics.

	Kolmogorov–Smirnov			Skewness	Kurtosis	Min	Max	Mean	Ss
	Statistics	sd	*p*						
Family Life Subdimension	0.25	371.00	<0.01	−1.32	0.84	1.00	5.00	4.36	0.86
School Life Subdimension	0.11	371.00	<0.01	−0.54	−0.50	1.00	5.00	3.78	1.00
Personal/Social Life Subdimension	0.18	371.00	<0.01	−1.19	0.66	1.00	5.00	4.09	1.00
Social Media Addiction Scale for Adolescents	0.09	371.00	<0.01	0.60	−0.16	1.00	5.00	2.28	0.90

**Table 3:** Findings on the Comparison of Scale Scores Based on Demographic Characteristics.

		n	Family Life Subdimension Scores	School Life Subdimension	Personal/Social Life Subdimension	Social Media Addiction Scale for Adolescents
Gender	Boy	90	4.48 ± 0.72	3.64 ± 0.98	4.19 ± 0.83	2.08 ± 0.81
	Girl	281	4.32 ± 0.90	3.82 ± 1.00	4.06 ± 1.04	2.35 ± 0.92
			t: 1.46; *p*: 0.15	t: −1.54; *p*: 0.13	t: 1.04; *p*: 0.30	t: −2.55; *p*: 0.01
Time spent on social media	0–2 h ^1^	109	4.48 ± 0.73	3.94 ± 1.03	4.17 ± 0.91	1.77 ± 0.79
	2–4 h ^2^	107	4.44 ± 0.89	3.85 ± 0.95	4.10 ± 1.03	2.11 ± 0.72
	4–6 h ^3^	116	4.21 ± 0.92	3.51 ± 0.92	4.02 ± 1.04	2.68 ± 0.81
	6 + h ^4^	39	4.24 ± 0.90	3.93 ± 1.11	4.07 ± 1.01	3.02 ± 0.87
			F: 2.38; *p*: 0.07	F: 4.29; *p*: 0.01 Difference: 1 > 3	F: 0.42; *p*: 0.74	F: 38.09; *p*: 0.00 Difference: 1 < 2.3, 4 ve 2 < 3.4

^1, 2, 3, 4^: Range of hours spent on social media; t: Independent Samples *t*-test; F: One-Way Analysis of Variance (ANOVA).

**Table 4:** Findings on the Prediction of Social Media Addiction Scores for Adolescents.

Dependent Variable: Social Media Addiction Scale for Adolescents									
	B	Stand. Deviation	Beta	t	*p*	CI Sub	CI Upper	r	VIF
Constant	2.25	0.27		8.36	<0.01	1.72	2.78		
Family Life Subdimension	0.06	0.05	0.05	1.06	0.29	−0.05	0.16	0.14	1.30
School Life Subdimension	0.15	0.04	0.17	3.37	<0.01	0.06	0.24	0.20	1.23
Personal/Social Life Subdimension	−0.04	0.05	−0.04	−0.74	0.46	−0.06	0.13	−0.08	1.46
Gender = (Girl) Reference Category: Boy	0.27	0.09	0.13	2.87	<0.01	0.09	0.46	0.13	1.03
Time spent on social media: 2–4 h Reference Category: 0–2 h	0.35	0.11	0.17	3.29	<0.01	0.14	0.55	−0.12	1.42
Time spent on social media: 4–6 h	0.84	0.10	0.43	7.98	<0.01	0.63	1.04	0.30	1.48
Time spent on social media: 6 h+	1.22	0.14	0.42	8.48	<0.01	0.94	1.51	0.28	1.22
**F(7.363) = 20.03 *p*: <0.01 R: 0.279 Düz. R: 0.265 Durbin–Watson: 2.1**									

CI: Confidence Interval; r: Correlation Coefficient.

## Data Availability

The original contributions presented in this study are included in the article. Further inquiries can be directed to the corresponding author.
